# The Illusion of Knowing in Metacognitive Monitoring: Effects of the Type of Information and of Personal, Cognitive, Metacognitive, and Individual Psychological Characteristics

**DOI:** 10.5964/ejop.v14i2.1418

**Published:** 2018-06-19

**Authors:** Maria Mykolaivna Avhustiuk, Ihor Demydovych Pasichnyk, Ruslana Volodymyrivna Kalamazh

**Affiliations:** a Department of International Relations, National University of Ostroh Academy, Ostroh, Ukraine; b Department of Psychology and Pedagogy, National University of Ostroh Academy, Ostroh, Ukraine; Department of Psychology, Webster University Geneva, Geneva, Switzerland; University of Neuchâtel, Neuchâtel, Switzerland

**Keywords:** the illusion of knowing, metacognitive monitoring, learning activity, metacognitive judgements, overconfidence, underconfidence, the illusion of not knowing

## Abstract

The aim of the paper is to analyse the illusion of knowing in metacognitive monitoring of the learning activity of university students (n = 262). The analysis focuses on the effects of the different types of information proposed and of personal, cognitive, metacognitive, and individual psychological characteristics of the participants. The research has shown that the illusion of knowing can occur in all types of metacognitive judgments, but is more evident in prospective judgments and depends on the type of information, its length and style, task type, etc. There are empirically established correlations between the selected personal, cognitive, and metacognitive characteristics. Gender and age differences in the manifestation of the illusion of knowing are not observed, although it is found that women tend towards overconfidence. The results also showed that the illusion of knowing is more typical for younger students, especially for those with lower levels of academic achievements.

## Metacognitive Monitoring Concept

Metacognitive monitoring is viewed as the way of checking students’ cognitive activity and how these results direct to the solution of certain cognitive tasks, such as recalling answers, doing tests, and reading texts (Savin & Fomin, 2013). It is also viewed as human evaluation of his/her own knowledge, knowledge of cognitive strategies, and knowledge of conditions that affect the learning process (Koriat, 1993; Valdez, 2013); or as explicit judgements that facilitate development of cognitive processes (Serra & Metcalfe, 2009). Separate aspects of metacognitive monitoring reliability are studied by Maki and Berry (1984) (metacomprehension of text material), Epstein, Glenberg, and Bradley (1984) (contribution of text variables to the illusion of knowing), Nelson and Narens (1990) (metamemory), Koriat (1993, 1997) (the accessibility model of the feeling of knowing; a cue-utilization approach to judgements of learning), Pulford (1996) (overconfidence in human judgements), Ilyina (2003) (learning motivation), Nietfeld et al. (2005) (metacognitive monitoring accuracy and student performance), Karpov and Skitiaeva (2005) (metacognitive processes of personal identity), Dubovitskaia (2005) (learning motivation), Moore and Cain (2007) (overconfidence and underconfidence), Zabrucky, Lin, and Agler (2008) (metacognition and learning), Parkinson (2009) (metacognition and word learning), Schraw (2009) (conceptual analysis of metacognitive monitoring), Savin and Fomin (2013) (cognitive aspects of education), Dotsevych (2013) (diagnosis of metacognitive competence).

Review of the results of theoretical analysis of the issue helped us group the types of metacognitive monitoring according to criteria of reliability (accurate and inaccurate monitoring), level of performance (local and global monitoring), temporal implication (on-line and off-line monitoring), learning achievements (subject-specific and general monitoring), cognition plot (monitoring of comprehension, monitoring of metamemory, and monitoring of performance), level of understanding (analytical (explicit) and non-analytical (implicit) monitoring), basis of judgements (information-based and experience-based monitoring) (Avhustiuk, 2016).

Correlations of subjective and objective success of task performance make it possible to establish metacognitive monitoring reliability and to highlight its errors. These errors are often manifested as two phenomena: overconfidence (the illusion of knowing) and underconfidence (the illusion of not knowing).

## The Illusion of Knowing Phenomenon

The first studies of the illusion of knowing were conducted by Glenberg, Wilkinson, and Epstein (1982), who showed it as the belief that comprehension has been attained, when, in fact, comprehension was not achieved. The phenomenon can occur during text learning when people express too unreasonably erroneous beliefs that information has been acquired (Dunlosky, Rawson, & Middleton, 2005; Glenberg, Wilkinson, & Epstein, 1982), or while studying pairs of words, as people often tend to overestimate the likelihood that they will later recall them (Eakin, 2005). Typically, this illusion appears in judgements made immediately after the learning process because assimilated information is still in the working memory (Nelson & Dunlosky, 1991).

In the psychological literature it is difficult to find some proper explanations for the causes and mechanisms of the illusion of knowing. The illusion of knowing is usually viewed as subjective overconfidence in the correctness of learning and understanding the information that objectively is incorrect (Commander & Stanwyck, 1997; Dunlosky, Rawson, & Middleton, 2005; Epstein, Glenberg, & Bradley, 1984; Glenberg & Epstein, 1985; Glenberg & Epstein, 1987; Glenberg, Wilkinson, & Epstein, 1982; Kroll & Ford, 1992); overconfidence in the correctness of task performance (Begg et al., 1996; Fazio & Marsh, 2008; Kelley & Lindsay, 1993; Kolers & Palef, 1976; Nelson & Dunlosky, 1991; Nelson & Narens, 1990; Nelson, Narens, & Dunlosky, 2004; Parkinson, 2009); or overconfidence in the ability to remember information that cannot be remembered (Castel, McCabe, & Roediger, 2007; Eakin, 2005; Koriat & Bjork, 2005).

The illusion of knowing is preceded by the illusions of competence (Bjork, 1999; Castel, McCabe, & Roediger, 2007; Dunning et al., 2003; Koriat & Bjork, 2005; Metcalfe, 1998; Zechmeister & Shaughnessy, 1980), the illusions of remembering (Jacoby, Bjork, & Kelley, 1994), the illusions of familiarity (Whittlesea, 1993), and the illusions of understanding (Bjork, 1999; Epstein, Glenberg, & Bradley, 1984; Glenberg, Wilkinson, & Epstein, 1982; Jee, Wiley, & Griffin, 2006). The most common reason for these illusions is inaccurate calibration. A number of studies (Jacoby, Bjork, & Kelley, 1994; Whittlesea, 1993) have found that it is almost impossible to avoid such illusions because people do not feel or notice them and continue erroneously proving the correctness of their decisions.

Alongside the term of the illusion of knowing such terms as cognitive optimism (Metcalfe, 1998), overconfidence (Eakin, 2005; Gigerenzer, Hoffrage, & Kleinbölting, 1991; Moore & Healy, 2008; Pulford, 1996), and subjective overconfidence in self-knowledge (Savin & Fomin, 2013) are also used. Such inconsistency of terminological apparatus complicates the comparison of the research results. Metcalfe (1998) believes that the basis of the illusion of knowing, or so-called ‘cognitive optimism’, is self-deception that can seem to optimize different learning activities. People become aware that their answers are wrong, but they convince themselves in contrary arguments. The reason for their conviction is that they have high levels of cognitive ability to recall information. A number of scientists show that the illusion of knowing is influenced by two phenomena: failure to identify contradictions (Epstein, Glenberg, & Bradley, 1984; Glenberg, Wilkinson, & Epstein, 1982) and overestimation of understanding level (Glenberg & Epstein, 1987; Glenberg, Wilkinson, & Epstein, 1982; Jacoby, Bjork, & Kelley, 1994; Kelley & Lindsay, 1993). These concepts are very controversial, and it is difficult to find out whether any of them can be a single cause of false subjective confidence. It has been suggested that successful determination of contradictions is not a guarantee that the illusion of knowing will not occur (Pulford, 1996).

We consider the illusion of knowing as a metacognitive monitoring error resulting from subjective overconfidence in knowing that does not meet objective success of task performance. The illusory knowledge effect that occurs afterwards is understood as knowledge that is incomplete, distorted, disfigured, undistinguished between ‘already known’ and ‘just learned’, unstructured, uncomprehended because of the illusion of knowing (Avhustiuk, 2016).

## Overconfidence, Underconfidence, and Hard-Easy Effect

Overconfidence can occur when confidence ratings of metacognitive judgements are higher than the levels of actual performance. It means that overconfidence is caused by excessive self-confidence in believing that one knows what one does not really know (Bradshaw, 2000; Gigerenzer, Hoffrage, & Kleinbölting, 1991; Jacoby, Bjork, & Kelley, 1994; Kvidera & Koutstaal, 2008; McKenzie, 1997; Pulford, 1996). Overconfidence can be of various types: overestimation of one’s actual performance (Dunning et al., 2003; Moore & Healy, 2008), overplacement of one’s performance relative to others (Moore & Healy, 2008), and excessive correctness of one’s beliefs (Moore & Healy, 2008). In general, scientists highlight two possible reasons for overconfidence: errors of the external nature that appear as a result of limitation in knowledge, and errors of information in the processing system or so called errors of the inner nature (Juslin, Winman, & Olsson, 2000).

Underconfidence also negatively affects monitoring efficiency of learning and understanding. Among the reasons for underconfidence there may be inaccurate methods used to evaluate future results or excessively large amount of important information that is confused with the less important. This results in an inappropriate performance and lack of self-confidence (Fajfar & Gurman, 2009; Griffin & Tversky, 1992; Hacker, Bol, & Keener, 2008; Kvidera & Koutstaal, 2008). Uncertainty in successful performance of simple tasks often leads to a situation when a person can spend much more time processing already learned material, and much less time and efforts studying issues that really require more attention (Fajfar & Gurman, 2009). Nevertheless, the underconfidence effect is not a potential threat in the process of learning. It rather provokes people into control and repetition of the learned information (Fajfar & Gurman, 2009).

A hard-easy effect is another manifestation of the illusion of knowing. It concerns such characteristics of the learned material as task complexity and structure. People tend to underestimate their actual performances on easy tasks, and to overestimate them on difficult tasks. As task complexity increases, the number of correct answers decreases, but people’s confidence in the correctness of their answers increases (Flannelly & Flannelly, 2000; Griffin & Tversky, 1992; Moore & Healy, 2008). According to Moore and Healy (2008), “on difficult tasks people overestimate their actual performances but also mistakenly believe that they are worse than others”. When the level of performance is high, it is underestimated, whereas when it is low, it is overestimated (Flannelly & Flannelly, 2000; Griffin & Tversky, 1992; Juslin, Winman, & Olsson, 2000; Klayman et al., 1999; Merkle, 2009; Moore & Healy, 2008).

## Effects of the Type of Information and of Personal, Cognitive, Metacognitive, and Individual Psychological Characteristics

The results of the theoretical analysis show that the processes of metacognitive monitoring of students’ knowledge, learning skills, memory capacity, and tasks performance are correlated with different types of information and with personal, cognitive, metacognitive, and individual psychological characteristics (e.g., age and gender).

A significant role is played by the characteristics of information. To this group we include type and style of information, its content and length, level of task complexity, task type, etc.

Many scientific works demonstrate that in metacognitive monitoring reliability a significant role is played by different cues and heuristics (Koriat, 1997; Serra & Metcalfe, 2009). Detailed descriptions of the cues and heuristics that facilitate and impede implementation of metacognitive monitoring judgements in the learning activity are presented by Koriat (1997), Koriat and Levy-Sadot (2001), Koriat, Nussinson, Bless, and Shaked (2008), and Serra and Metcalfe (2009). In particular, Koriat (1997) identifies three classes of these cues: intrinsic, extrinsic, and mnemonic, which depend on number, complexity, semantic content, and conditions of processing the learned information. These cues do not always have a positive impact on metacognitive judgements as they sometimes can be ignored (Dunlosky & Nelson, 1992; Koriat, 1997) or misunderstood (Benjamin, Bjork, & Schwartz, 1998).

Some scientists study metacognitive monitoring reliability in the process of reading texts (Glenberg, Wilkinson, & Epstein, 1982), while others study its role in the process of words (Eakin, 2005; Parkinson, 2009) and statements learning (Kolers & Palef, 1976; Nelson & Narens, 1980; Smith & Clark, 1993). The level of task complexity significantly influences metacognitive monitoring judgements of the learning accuracy. These results are due to Brown (1987), Pulford (1996), Kruger and Dunning (1999), and others. Information context, its informativity, interesting representation, and value also play important roles in metacognitive monitoring reliability (Koriat et al., 2014).

Pallier et al. (2002) found slight relations between task content and overconfidence. Hacker, Bol, and Bahbahani (2008) demonstrated that higher levels of calibration are possible only in accordance with higher levels of knowing of the context of information.

It is necessary to conduct an investigation of the illusion of knowing in the sphere of information style as scientific provements of these correlations are not sufficient. Information length, ease of access, and additional general information also influence metacognitive judgements of learning (Commander & Stanwyck, 1997; Koriat, 1993; Koriat, Lichtenstein, & Fischhoff, 1980; Pulford, 1996). Thus, Commander and Stanwyck (1997) have shown that the illusion of knowing is more dependent on smaller texts, while larger texts, on contrary, can provide metacognitive monitoring reliability.

It is rather difficult to study factors of metacognitive reliability without taking into account task type. According to Koriat, Lichtenstein, and Fischhoff (1980), the reason of systematic errors in metacognitive judgements is a tendency to choose positive answers, and to ignore the answers that do not coincide with personal beliefs. Pallier et al. (2002) and de Carvalho Filho (2009) showed that open-answer questions provide higher levels of metacognitive monitoring reliability in the process of knowledge self-esteem as compared with multiple-choice questions. Dutke, Barenberg, and Leopold (2010) are convinced that knowing the task type before doing tests can improve metacognitive monitoring reliability. According to Savin and Fomin (2013), systematic performing of tasks of the same type can lead to inadequate metacognitive monitoring.

Some scientists study metacognitive monitoring reliability in the context of such factors as rereading (Dunlosky & Rawson, 2012; King, Zechmeister, & Shaughnessy, 1980; Koriat & Bjork, 2006) and generalization (Begg et al., 1996; Koriat, 1997). Some attempts have been made (Ward & Clark, 1989) to study such factors of metacognitive monitoring reliability as internal and external feedback. According to the scientists, when people systematically analyse assessments of their confidence in the correctness of doing tasks, they can achieve greater objective success of task performance.

The psychological literature data demonstrate that metacognitive monitoring processes have tight connections with personal characteristics such as intellectual level, self-monitoring capacity, necessity to understand task performance, persistence, activity, and positive emotions. (Pallier et al., 2002). There are also personal factors that influence overconfidence such as motivation and self-esteem, and individual psychological differences (e.g., age, gender, character) (Pulford, 1996).

Ilyina (2003) has studied the structure of motivation in the learning activity of university students and highlighted its three main goals: to receive knowledge, to have an occupation, and to get a diploma. Romek (1998) provides diagnosis of confidence evaluation. This approach is seen as a generalized method of positive self-assessment of human skills and abilities. Karpov and Skitiaeva (2005) study reflexivity and highlight its three main types: situational (active), retrospective, and prospective reflexivity.

An important role in metacognitive monitoring reliability is also played by cognitive characteristics. Academic achievements should be taken into account. According to Pallier et al. (2002), Jee, Wiley, and Griffin (2006), and Savin and Fomin (2013), people with higher levels of knowledge tend towards lesser overconfidence. Unsuccessful students, in their turn, learn material quickly and not thoughtfully, do not stop on problematic aspects, and do not take into account misunderstood parts (Winne, 2010).

Some scientists study influence of the level of intellect (Koriat, 2012; Kornilova et al., 2008; Miele & Molden, 2010; Miller & Geraci, 2011) and self-efficacy (Schwarzer, Jerusalem, & Romek, 1996) on metacognitive monitoring reliability. Thus, according to these and some other researchers, cognitive characteristics include specific degree of knowledge, prior learning experience, self-efficacy, implicit theories (fixed/changeable intellect) (Kornilova et al., 2008), and also students’ academic achievements.

Diagnostic methods of recent years also allow for consideration of the role of metacognitive characteristics such as metacognitive knowledge and metacognitive activity (Kashapov, 2012), as well as metacognitive awareness (Schraw & Dennison, 1994). A method of diagnosis of metacognitive knowledge and metacognitive activity is used to study metacognitive characteristics. According to this method, metacognitive knowledge is understood as human knowledge about means of receiving and processing information, knowledge about types and content of tasks, and knowledge about metacognitive strategies in problem solving. Metacognitive activity is a process of obtaining and selecting information, its control, change, and metacognition planning (Kashapov, 2012). Metacognitive awareness, or so called metacognitive involvement in the learning activity, acts as a specific degree of metacognitive monitoring skills formation in cognitive activity of the learning process (Schraw & Dennison, 1994).

It is difficult to establish metacognitive monitoring reliability factors without studying individual psychological characteristics. Gender differences and age peculiarities were among the primarily concerns taken into account in our group study. Analysis of psychological and educational literature has also shown that metacognitive monitoring reliability cannot be studied apart from appropriate psychological and pedagogical conditions.

## Objectives

The research is centred in a precise theoretical framework of the illusion of knowing in metacognitive monitoring. In particular, we aim to study this phenomenon in terms of investigating its influence on metacognitive monitoring reliability of the learning activity of university students. In our previous work (Pasichnyk, Kalamazh, & Avgustiuk, 2017) we introductorily aimed to clarify the illusion of knowing in the educational activity in terms of reliability of metacognitive monitoring accuracy factors and to find out some correlations between specified psychological characteristics providing brief analysis of their impact on the occurrence of the illusion of knowing. In the current paper we thoroughly study the effects of such factors as different types of the learned information and also personal, cognitive, metacognitive and individual psychological characteristics of the participants. Moreover, we set a goal to provide the detailed analysis of the impact of the highlighted characteristics on the illusion of knowing. So, specifically, the main aims of the present study are: to investigate the grouped characteristics of metacognitive monitoring reliability and to thoroughly analyze their impact on the learning activity of university students; to provide the study of the empirical results of the peculiarities of the illusion of knowing in metacognitive monitoring; to analyze the peculiarities of the illusion of knowing according to the theoretical analysis and empirical investigation of the phenomenon.

## Method

### Participants

A total of 262 university students of different faculties of the National University of Ostroh Academy (Ukraine) (192 female and 70 male students, *M* = 19.5; *SD* = 1.87) voluntarily participated in this study for free. All participants were students in their 1^st^ to 5^th^ year of university. The sample consisted of Ukrainian students only.

### Materials

In general, the study was conducted in two stages: a diagnostic stage and a laboratory experiment stage. At the diagnostic stage the participants were asked to answer questions from a questionnaire aiming to ascertain psychological characteristics of students that according to the results of theoretical analysis were related to metacognitive monitoring reliability (see Pasichnyk, Kalamazh, & Avgustiuk, 2017). Empirical reference of the level of students’ knowledge was studied with the help of the generalization of their academic achievements during semester.

To diagnose personal characteristics we used such questionnaires as a method of motivation diagnosis (Ilyina, 2003); a method of self-confidence diagnosis (Romek, 1998); a method of reflexivity diagnosis (Karpov & Skitiaeva, 2005). To diagnose cognitive characteristics we used a self-efficacy assessment test (Schwarzer, Jerusalem, & Romek, 1996); a method of implicit theories diagnosis (according to Dvek’s questionnaire) (Kornilova et al., 2008). To study metacognitive characteristics we used a diagnosis method of metacognitive involvement in the learning activity (Schraw & Dennison, 1994); a method of diagnosis of metacognitive knowledge and metacognitive activity (Kashapov, 2012). Also at this stage a sample test was carried out to study normal distribution of equivalence and the highlighted characteristics.

At the stage of the laboratory experiment 6 texts, 18 statements, and 18 pairs of words in Ukrainian were chosen as a stimuli material needed to be learned. The texts were of different styles (the scientific prose, the newspaper and the belletristic styles) and of different length (larger text volume accounted 25-30 sentences and smaller text volume accounted 10-15 sentences). The participants read two texts of the same style according to different length. All quantitative data were divided into nine groups depending on such factor as task type: open-answer questions for texts, statements, and word pairs; ‘Yes’/‘No’/‘Do not know’ questions for texts, statements, and word pairs; multiple-choice questions for texts, statements, and word pairs. The aim of the pilot study was to standardize the stimulus material according to such criteria as the hard-easy effect, familiarity/unfamiliarity of information, and emotional impact.

### Procedure and Design

The participants had to learn 6 texts, 18 statements and 18 pairs of words in Ukrainian. They performed prospective metacognitive judgements of learning about confidence (JOLs) and prospective judgements about the number of correct answers (aJOLs), as well as similar retrospective metacognitive judgements of both types (RCJs and aRCJs). With the help of proper calibration procedure we defined average indicators of the illusion of knowing (overconfidence) and average indicators of the illusion of not knowing (underconfidence).

**Figure 1:**
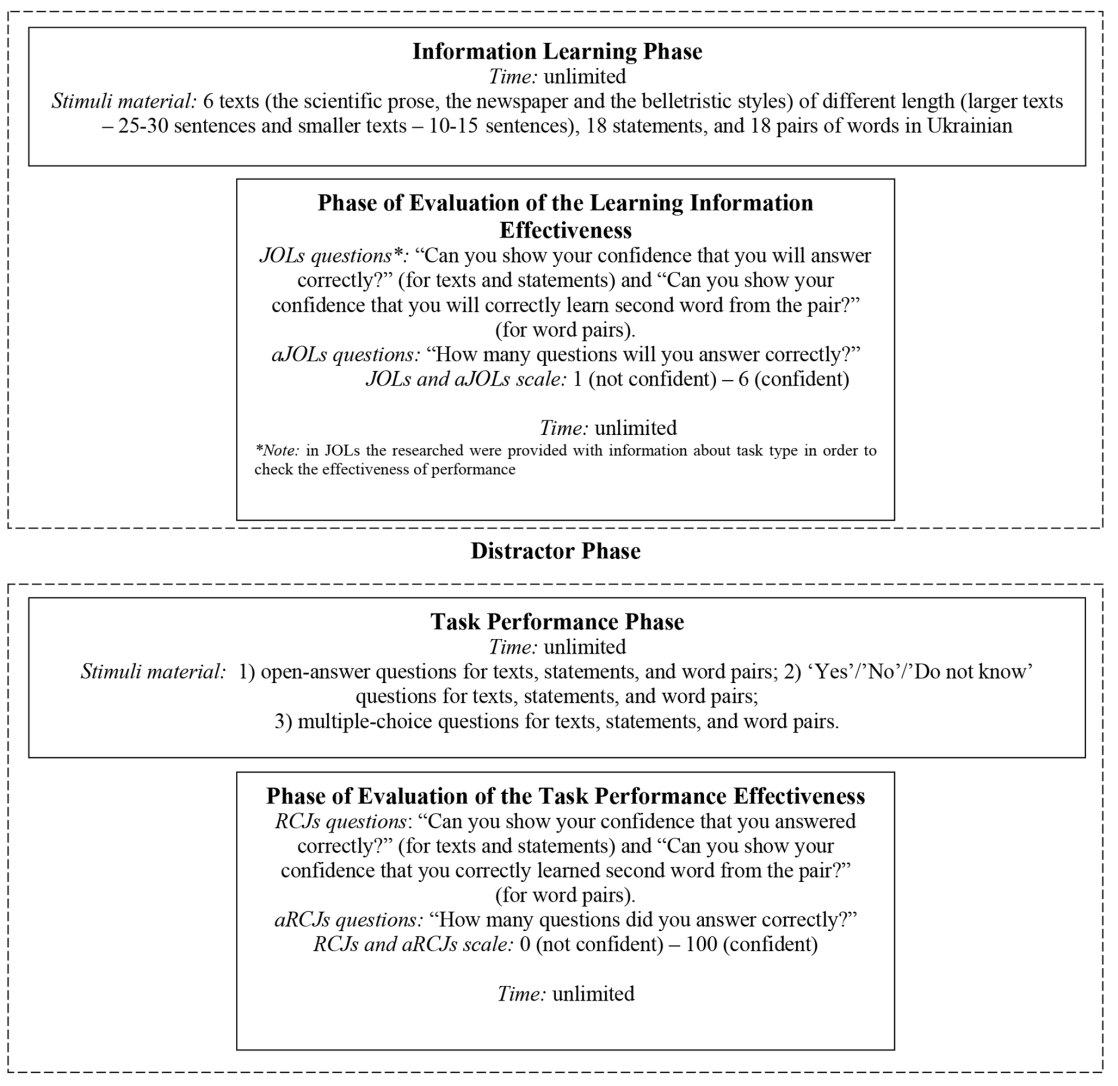
Scheme of the experiment.

In general, the experiment consisted of the following phases: an Information Learning Phase, a Phase of Evaluation of the Learning Information Effectiveness, a Distractor Phase (served as a possibility for the participants to rest doing non-evaluated activity), a Task Performance Phase, and a Phase of Evaluation of the Task Performance Effectiveness. Experimental scheme is presented in Figure 1.

During the Information Learning Phase students learned 6 texts, 18 statements, and 18 pairs of words. The Phase of Evaluation of the Learning Information Effectiveness served as the possibility to study subjective confidence in the correctness of learning. With the help of a scale from 1 (absolutely unconfident) to 6 (absolutely confident) students performed prospective metacognitive judgements of learning about confidence (JOLs). Moreover, prospective judgements about the number of correct answers (aJOLs) were also performed. During the Task Performance Phase students answered the needed questions that were used to find the learning levels of the given information. They answered open-answer questions, ‘Yes’/‘No’/‘Do not know’ questions, and multiple-choice questions for texts, statements, and word pairs. The Phase of Evaluation of Task Performance Effectiveness served as the possibility to evaluate the level of correctness of information learning and task performing. Again, with the help of the scale from 1 to 6 students performed retrospective metacognitive judgements of learning about confidence (RCJs) and retrospective judgements about the number of correct answers (aRCJs).

### Analysis

All the received data were processed by a computer program *IBM SPSS Statistics 20* and calculations were done by *Excel* program. Data were processed by means of mathematical and statistical methods such as *ANOVA* analysis, *T*-test, correlation coefficient of Goodman-Kruskal, Spearman rank of correlation, Pearson linear correlation, *O/U* index, and calibration index (see Pasichnyk, Kalamazh, & Avgustiuk, 2017).

Metacognitive monitoring errors we aimed to find (overconfidence as the illusion of knowing and underconfidence as the illusion of not knowing) were determined as the difference between subjective evaluation of the accuracy of retrieval (metacognitive judgements rating) and the observed reproduction (relative share of results according to total number of tasks). The larger the difference is, the greater is the manifestation of the illusion of knowing, and vice versa (Gigerenzer et al., 1991; Ward & Clark, 1989). To do this we used a three-level scale ​​from -1 to +1 (for more detailed description see Pasichnyk, Kalamazh, & Avgustiuk, 2017).

## Results

The results of the received data are described according to the divided groups of metacognitive monitoring reliability factors. These are different types of information and also personal, cognitive, metacognitive, and individual psychological characteristics of the participants.

### The Illusion of Knowing Levels

The results from the diagnostic stage showed predominance of learning motivation to gain knowledge (48.7%) and skills development (39.2%), middle (49%) and high (29.5%) levels of general self-confidence, self-efficacy (middle level – 44.3%, high level – 32.6%), and metacognitive awareness (middle level – 43.5%, high level – 35.5%), as well as middle levels of metacognitive knowledge (62.8%) and metacognitive activity (58.7%). The research data also showed a great amount of students with middle levels of reflexivity (56.7%), and significant number of them showed low-reflection (30.9%). In general, the results of the study showed that 59.4% of the participants committed errors in JOLs, and the majority of them (31.3%) showed overconfidence in task performance correctness. Moreover, 50% of the students committed metacognitive monitoring errors in the course of aJOLs, while 35.9% of the students were overconfident in task performance correctness. At the same time, the average results of the illusion of knowing were slightly different in JOLs (*M*_JOL_ = .27, *SD* = .61) and in aJOLs (*M*_aJOL_ = .25, *SD* = .69) (*p* ≤ .05). The number of students who showed the illusion of knowing was not significantly different. It can mean that before task performance overconfidence is not significantly dependent on task type. However, in RCJs there was a decrease (6.3%) of students’ overconfidence in task accuracy, and in aRCJs the decrease reached 11.7%. Average value of overestimation remained unchanged.

aRCJs were the most accurate as 61% of the students who took part in the study showed adequate accuracy levels of metacognitive monitoring (*M*_aRCJ_ = .01, *SD* = .18, *p* = .05). In aJOLs and aRCJs the proportion of those who overestimated the number of correctly performed tasks was significantly higher in comparison with those students who showed underestimation. However, among those students who underestimated the number of correctly performed tasks, the indicators of the illusion of not knowing were the highest (*M*_aJOL_ = -.37, *SD* = .41, and *M*_aRCJ_ = -.33, *SD* = .48) (*p* ≤ .05).

The illusion of knowing mostly occured in aJOLs (35.9%). Before tasks performance among those students who underestimated possible number of correctly performed tasks the degree of the illusion of not knowing was the highest (*M*_aJOL_ = -.37, *SD* = .41, *p* = .05) as after task performance the accuracy of judgements significantly increased. *T*-test for pair samples showed significant differences in the rates of errors in metacognitive judgements between JOLs and aJOLs (*t*(56) = 2.09, *p* ≤ .05), between aRCJs and RCJs (*t*(56) = 2.23, *p* ≤ .05), and between JOLs and RCJs (*t*(56) = 2.09, *p* ≤ .05). In retrospective judgements of both types metacognitive monitoring accuracy was higher. According to the results, those students who made mistakes in monitoring reduced the proportion of those who showed the illusion of knowing. The results relate to the research data of Busey et al. (2000), McCormick (2003), Nelson (1999), Thiede and Dunlosky (1994), and others, that found that overconfidence leads to the illusory feeling of knowing in retrospective monitoring judgements.

### Correlations Between Personal, Cognitive, and Metacognitive Characteristics

According to the empirical results, there were found correlations between the studied personal, cognitive, and metacognitive characteristics of students. Learning motivation positively correlated with self-confidence (*r* = .17, *p* = .05), reflexivity (*r* = .43, *p* = .01), metacognitive awareness (*r* = .31, *p* = .01), metacognitive knowledge (*r* = .22, *p* = .05), and metacognitive activity (*r* = .26, *p* = .01). Self-confidence also positively correlated with self-efficacy (*r* = .44, *p* = .01), metacognitive awareness (*r* = .27, *p* = .01), metacognitive knowledge (*r* = .31, *p* = .01), and metacognitive activity (*r* = .19, *p* = .05). Reflexivity also correlated with metacognitive awareness (*r* = .35, *p* = .01). The correlations between self-efficacy and the implicit theory of intellect (*r* = .24, *p* = .01), metacognitive awareness (*r* = .34, *p* = .01), and metacognitive knowledge (*r* = .26, *p* = .01) were viewed as well. Metacognitive awareness in addition to the correlations with learning motivation, self-confidence, reflexivity, and self-efficacy, also positively correlated with metacognitive knowledge (*r* = .36, *p* = .01). Direct correlations (Pearson correlation) between the indicators of the illusion of knowing in prospective (*r*_JOLs_ = -.21, *p* = .05) and retrospective (*r*_RCJs_ = -.23, *p* = .01) judgements of learning were also found. Before task performance there were found close correlations between the indicators of the illusion of knowing and metacognitive activity (*r*_aJOL_ = -.18, *p* = .05), as well as metacognitive awareness (*r*_JOL_ = -.21, *p* = .05). In particular, significant correlations were found between the illusion of knowing and metacognitive activity, metacognitive awareness, and general self-confidence (also see Avhustiuk, 2016; Pasichnyk, Kalamazh, & Avgustiuk, 2017).

### Effects of the Type of Information

**Figure 2:**
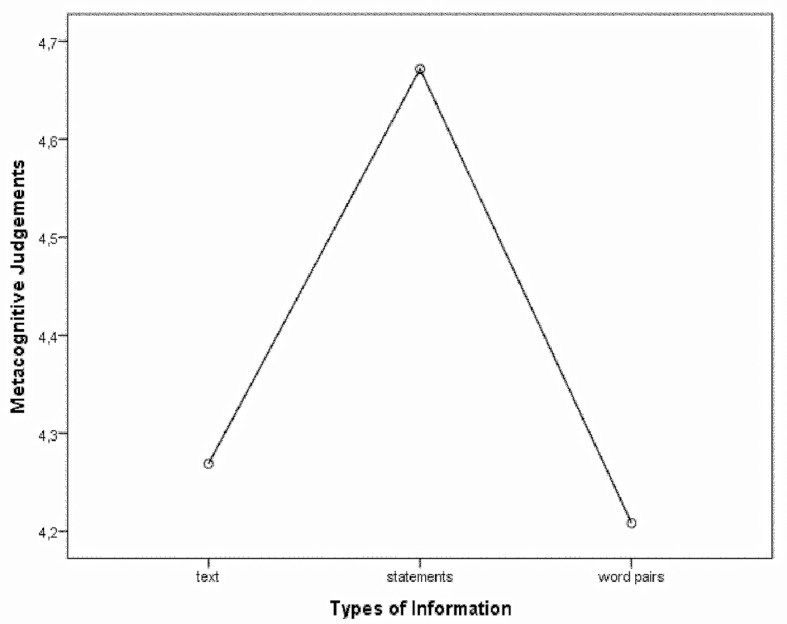
Performance rankings of metacognitive judgements in terms of the type of information.

According to *ANOVA* analysis, statistically significant differences were found in the average values ​​of the studied judgements of learning about confidence (RCJs) and the type of information [*F*(2, 56) = 17.78, *p* < .001]. The highest level of overconfidence was shown when performing the statements (*M* = 4.67, *SD* = 1.59, *p* < .001), whereas significantly lesser confidence was observed while reading texts (*M* = 4.27, *SD* = 1.53, *p* < .001), and the lowest level of confidence appeared in learning of word pairs (*M* = 4.21, *SD* = 1.9, *p* < .001). The results are presented in Figure 2.

### Effects of the Text Style and Length

Students showed higher ratings of metacognitive judgements while memorising texts of the belletristic style (*M* = 4.69, *SD* = .75, *p* = .04) compared to the texts of the newspaper style (*M* = 4.44, *SD* = 2, *p* = .05) and the style of the scientific prose (*M* = 4.43, *SD* = 2, *p* = .05).

**Figure 3:**
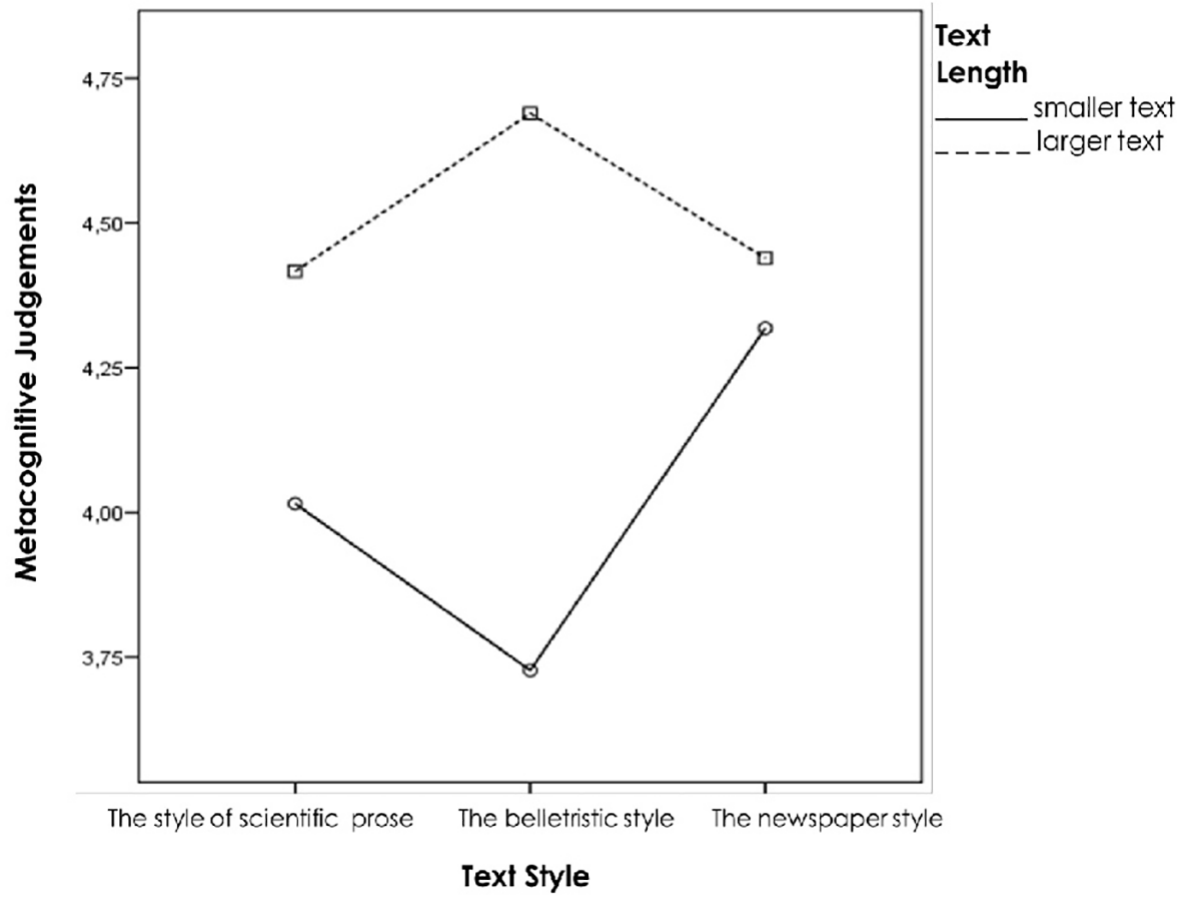
Performance rankings of metacognitive judgements in terms of text style and length.

Significantly higher confidence in the correctness of the learned material was shown while learning larger texts (*M* = 5.12, *SD* = .64, *p* = .04) in comparison with smaller texts (*M* = 3.5, *SD* = 1.88, *p* = .05). The same results were observed in terms of information style: students were more overconfident in their judgements of learning while working with larger text of the belletristic style (*M* = 4.69, *SD* = .75, *p* = .05), unlike while reading smaller text of the same style (*M* = 3.73, *SD* = 1.7, *p* = .05), also when working with larger texts of the scientific prose and of the newspaper styles (*M* = 4.43, *SD* = 2, *p* = .05, and *M* = 4.44, *SD* = 1.8, *p* = .05 respectively) if to compare with smaller texts of the same styles (*M* = 4.02, *SD* = 1.9, *p* = .05 – for the scientific style and *M* = 4.32, *SD* = 2.8, *p* = .05 – for the newspaper style). The results are presented in Figure 3.

### Effects of the Task Type

**Figure 4:**
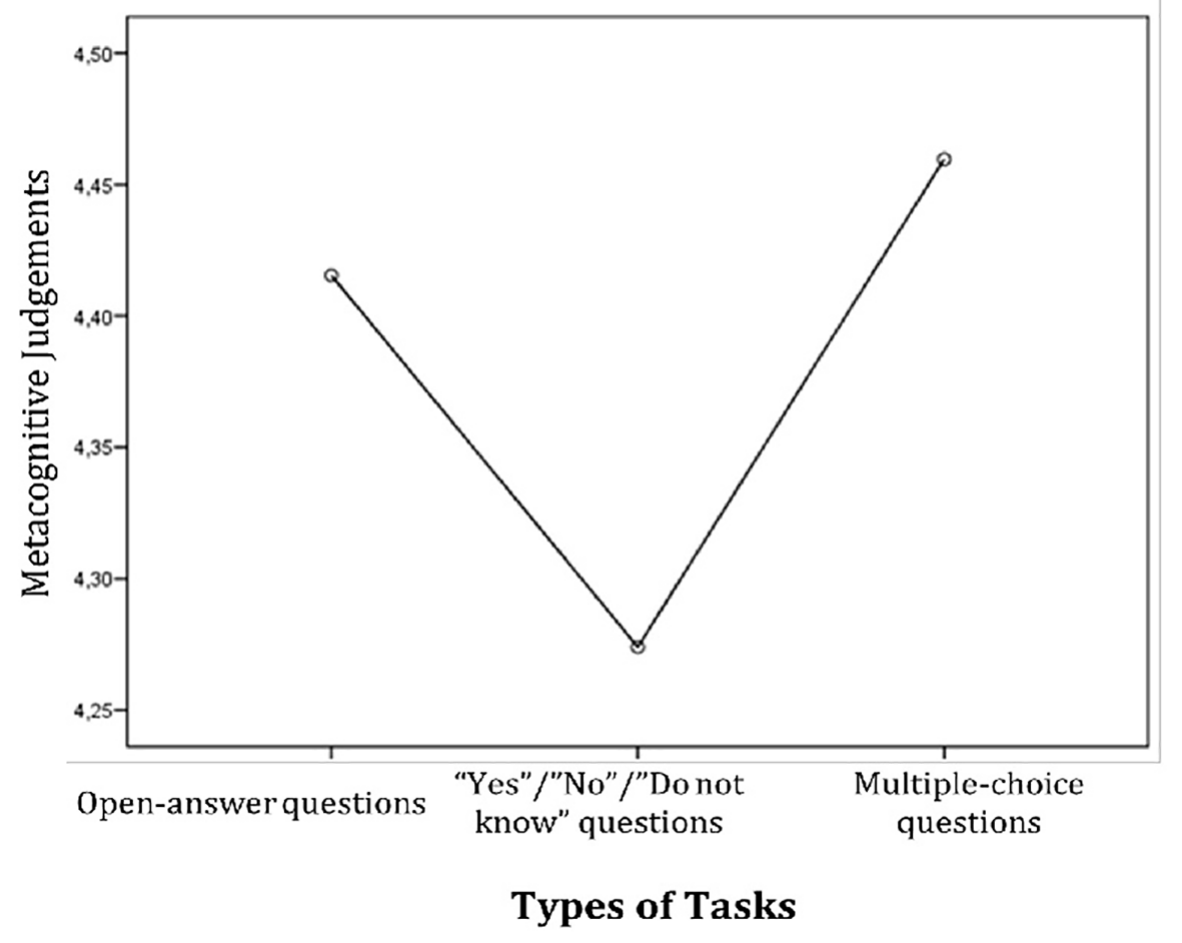
Performance rankings of metacognitive judgements in terms of task type.

The participants were more confident in the judgements of learning while answering multiple-choice questions (*M* = 4.46, *SD* = 1.66, *p* = .03), were less confident while answering open-answer questions (*M* = 4.42, *SD* = 1.71, *p* = .05), and showed the least levels of confidence while answering ‘Yes’/‘No’/‘Do not know’ questions (*M* = 4.28, *SD* = 1.69, *p* = .03). Students highly overestimated the accuracy of tasks performance that resulted in the illusion of knowing in multiple-choice questions for statements (*M*_O/U_ = .27, *SD* = .74, *p* = .01), and showed the greater accuracy in metacognitive judgements in open-answer questions for texts (*M*_O/U_ = .07, *SD* = .17, *p* < .001) and in ‘Yes’/‘No’/‘Do not know’ questions for texts (*M*_O/U_ = .09, *SD* = .13, *p* < .001). The results are presented in Figure 4.

### Metacognitive Monitoring Errors

Indicators of influence of the illusion of knowing were calculated with the help of a single factor analysis of variance and LSD-analysis. We found statistically significant differences between the average values ​​of the indicators of confidence index factors ‘open-answer questions for texts’ (*M* = .07; *SD* = .17, *p* < .001) and ‘multiple-choice questions for statements’ (*M* = .27, *SD* = .74, *p* < .001); between the average values ​​of the indicators of confidence index factors ‘open-answer questions for word pairs’ (*M* = .14, *SD* = .13, *p* = .01) and ‘multiple-choice questions for statements’ (*M* = .27, *SD* = .74, *p* ​​= .01). Statistically significant differences between the average values ​​of the indicators of confidence index factors ‘multiple-choice questions for statements’ (*M* = .27, *SD* = .74, *p* < .001) and ‘Yes’/‘No’/‘Do not know’ questions for texts’ (*M* = .1, *SD* = .14, *p* < .001) also occurred. Other differences were as follows: between the average values ​​of the indicators of confidence index factors ‘multiple-choice questions for statements’ (*M*_O/U_ = .27, *SD* = .74, *p* = .01) and ‘Yes’/‘No’/‘Do not know’ questions for word pairs’ (*M*_O/U_ = .12, *SD* = .16, *p* = .01); between the average values ​​of the indicators of confidence index factors ‘Yes’/‘No’/‘Do not know’ questions for statements’ (*M*_O/U_ = .27, *SD* = .74, *p* = .05) and ‘multiple-choice questions for texts’ (*M*_O/U_ = .13, *SD* = .11, *p* = .05); between the average values ​​of the indicators of confidence index factors ‘multiple-choice questions for statements’ (*M*_O/U_ = .27; *SD* = .74, *p* = .02) and ‘multiple-choice questions for texts’ (*M*_O/U_ = .14, *SD* = .13, *p* = .02); between the average values ​​of the indicators of confidence index factors ‘multiple-choice questions for statements’ (*M*_O/U_ = .27, *SD* = .74, *p* < .001) and ‘multiple-choice questions for word pairs’ (*M*_O/U_ = .14, *SD* = .17, *p* < .001). Average results of the illusion of knowing in metacognitive monitoring are presented in Table 1.

**Table 1:** Average Results of the Illusion of Knowing in Metacognitive Monitoring

Levels	*M* (*SD*)							
	Learning Motivation	Self-Confidence	Reflexivity	Intellect	Self-Efficacy	Metacognitive Knowledge	Metacognitive Activity	Metacognitive Awareness
aJOLs								
High	0.25 (0.19)	0.24 (0.19)	0.25 (0.18)	0.25 (0.1)	0.25 (0.14)	0.30 (0.24)	0.15 (0.1)	0.24 (0.21)
Middle	0.25 (0.2)	0.26 (0.17)	0.25 (0.19)	0.26 (0.13)	0.25 (0.47)	0.24 (0.12)	0.26 (0.17)	0.28 (0.16)
Low	0.21 (0.18)	0.19 (0.17)	0.25 (0.19)	0.18 (0.18)	0.15 (0.07)	0.21 (0.28)	0.25 (0.22)	–
aRCJs								
High	0.26 (0.15)	0.24 (0.2)	0.25 (0.2)	0.24 (0.17)	0.27 (0.09)	0.29 (0.14)	0.25 (.22)	0.25 (0.19)
Middle	0.23 (0.17)	0.26 (0.18)	0.27 (0.13)	0.30 (0.12)	0.23 (0.27)	0.23 (0.09)	0.25 (0.23)	0.20 (0.13)
Low	0.21 (0.28)	–	0.21 (0.14)	0.20 (0.23)	–	0.24 (0.19)	0.24 (0.2)	–
JOLs								
High	0.27 (0.3)	0.27 (0.18)	0.21 (0.17)	0.27 (0.12)	0.27 (0.18)	0.33 (0.27)	0.18 (0.09)	0.30 (0.19)
Middle	0.26 (0.2)	0.30 (0.21)	0.26 (0.17)	0.30 (0.31)	0.27 (0.18)	0.25 (0.08)	0.27 (0.21)	0.22 (0.42)
Low	0.19 (0.12)	0.25 (0.16)	0.30 (0.21)	0.19 (0.21)	0.24 (0.09)	0.23 (0.31)	0.28 (0.18)	–
RCJs								
High	0.26 (0.2)	0.24 (0.16)	0.30 (0.2)	0.25 (0.16)	0.28 (0.11)	0.28 (0.37)	0.26 (0.13)	0.25 (0.18)
Middle	0.25 (0.18)	0.28 (0.17)	0.26 (0.12)	0.26 (0.09)	0.23 (0.23)	0.24 (0.18)	0.24 (0.09)	0.24 (0.18)
Low	0.18 (0.14)	0.25 (0.2)	0.24 (0.16)	0.21 (0.2)	0.19 (0.08)	0.25 (0.17)	0.28 (0.1)	–

### Effects of Personal Characteristics

#### The Illusion of Knowing From the Spectrum of Learning Motivation

Those students who were focused on knowledge performed accurate metacognitive judgements. However, among the students targeted for profession who made accurate metacognitive judgements the accuracy of metacognitive monitoring was the highest (*M*_aJOL_ = -.006, *SD* = .01, *M*_aRCJ_ = -.006; *SD* = .02, *M*_JOL_ = .03, *SD* = .02, *M*_RCJ_ = .00, *SD* = .01) (*p* = .05). The research showed the correlations (Pearson criterion) between the indicators of self-confidence and the illusion of knowing in aRCJs (*r* = .32, *p* = .01) and RCJs (*r* = .24, *p* = .05).

#### The Illusion of Knowing From the Spectrum of Self-Confidence

The data for the scale of Romek’s methodology ‘self-confidence’ – ‘self-unconfidence’ showed that university students in the context of its various levels tend towards overconfidence as well as towards underestimation of the accuracy of tasks performance. Correlation analysis (Spearman criterion) showed the correlations between confidence indicators and the illusion of knowing in aRCJs (*r* = .32) and RCJs (*r* = .24) (*p* = .05).

#### The Illusion of Knowing From the Spectrum of Reflexivity

Highly reflexive students showed very high rates of underconfidence (*M* = -.74, *SD* = .27, *p* = .01) if compared with the participants with middle (*M* = -.42, *SD* = .22, *p* = .01) and low levels of reflexivity (*M* = -.47, *SD* = .17, *p* = .01). However, the performance of aRCJs among students with high and middle levels of reflexivity significantly increased the proportion of those who almost made no mistakes in metacognitive monitoring (from 58% to 73.7% and from 46.4% and 60.8% respectively).

### Effects of Cognitive Characteristics

#### The Illusion of Knowing From the Spectrum of the Implicit Theories of Fixed/Changeable Intellect

Analysis of variance *ANOVA* showed differences of the average values of the illusion of knowing according to the notions of fixed/changeable intellect, although they were not statistically significant. Results of the inner-group differences in the average values showed that in aJOLs and aRCJs, regardless of changeable intellect, the proportion of overconfident students dominated the proportion of underconfident students. The highest levels of underconfidence were shown by the students with the average levels of changeable intellect (*M* = -.53, *SD* = .21 and *M* = -.55, *SD* = .18 respectively) (*p* = .01).

#### The Illusion of Knowing From the Spectrum of Self-Efficacy

We found that the participants with middle and high levels of self-efficacy were more accurate in prospective and retrospective metacognitive judgements of learning rather than the participants with lower self-efficacy. The last demonstrated such error of metacognitive monitoring as the illusion of not knowing. The proportion of overconfident students in aJOLs and aRCJs (37% and 37,6% respectively) was much higher than the same proportion of underconfident students (10% and 14.3% respectively). Among the participants with lower levels of self-efficacy the proportion of underconfidence in JOLs was very high (55%), and the levels of the illusion of not knowing were also very high (*M* = .53, *SD* = .12, *p* = .01).

#### The Illusion of Knowing From the Spectrum of Academic Achievements

To determine the relations between the level of the illusion of knowing and academic achievements, semester overall results were analyzed. For more adequate results, average marks of each student were converted from a 100-scale to standard values of a 5-point scale (5 is the highest result). Results showed that the illusion of knowing was common for the participants with lower results of academic achievements.

### Effects of Metacognitive Characteristics

#### The Illusion of Knowing From the Spectrum of Metacognitive Knowledge and Metacognitive Activity

The results showed differences in terms of metacognitive knowledge between the indicators of the illusion of knowing in aJOLs and aRCJs [*F*(2, 56) = 3.38, *p* = .04] and differences in terms of metacognitive activity between the indicators of the illusion of knowing in aJOLs and aRCJs [*F*(2, 56) = 2.79, *p* = .07], as well in JOLs and RCJs [*F*(2, 56) = 3.21, *p* = .05]. There were also found statistically significant differences between the average values ​​of the indicators of the illusion of knowing in all kinds of metacognitive judgements of learning in metacognitive activity. The participants with lower levels of metacognitive activity showed the illusion of knowing (overconfidence) in all prospective and retrospective judgements of learning.

#### The Illusion of Knowing From the Spectrum of Metacognitive Awareness

There were found direct correlations (Pearson correlation) between the indicators of the illusion of knowing in prospective (*r*_JOL_ = -.21, *p* = .05) and retrospective (*r*_RCJ_ = -.23, *p* = .01) judgements of learning about confidence and performance indicators of metacognitive awareness. Before task performance there were found close correlations with the indicators of the illusion of knowing and metacognitive activity (*r*_aJOL_ = -.18, *p* = .05) and metacognitive awareness (*r*_JOL_ = -.21, *p* = .05). It was also found that among the participants with high and middle levels of metacognitive awareness a significant proportion of those students who almost did not commit errors in metacognitive monitoring notably increased (from 46.6% to 58% and from 56% to 74.8% respectively). The same trend was observed in the judgements of high and mid-reflexive students. The analysis data showed correlations between the levels of the illusion of knowing in all prospective (*r* = .21, *p* = .05) and retrospective metacognitive judgements (*r* = -.23, *p* = .01).

### Effects of Individual Psychological Characteristics

#### Gender Differences

In terms of individual psychological characteristics of the participants statistically significant differences between the illusion of knowing and gender peculiarities [*F*(2, 56) = .013, *p* = .99] were not found. It was fixed that women tended towards overconfidence in prospective and retrospective judgements of learning, although these levels were not high.

#### Age Peculiarities

Analysis of variance *ANOVA* showed statistically significant differences in terms of age peculiarities between the indicators of the illusion of knowing (*F*_aJOL_(2, 56) = 9.43, *F*_aRCJ_(2, 56) = 13.03, *F*_JOL_(2, 56) = 4.44, *F*_RCJ_(2, 56) = 6.95, *p* < .001). It can mean that the illusion of knowing in all kinds of prospective and retrospective judgements depends on age peculiarities. Moreover, we found that the participants of the age group of 17-19 were more overconfident (*M* = .06, *SD* = .19, *p* < .001), while the students of the age group of 20-22 tended towards underconfidence (*M* = -.41, *SD* = .47, *p* < .001).

## Discussion

The paper is devoted to the study of the illusion of knowing in metacognitive monitoring of the learning activity of university students. It allocates such factors of metacognitive monitoring reliability as different types of information, and also personal, cognitive, metacognitive, and individual psychological characteristics.

The findings presented here and in some other previous works (Pasichnyk, Kalamazh, & Avgustiuk, 2017) demonstrate that the illusion of knowing, regarded as overconfidence and an error of metacognitive monitoring, can occur in all types of metacognitive judgements. Nevertheless, it is more evident in prospective judgements. According to our results, subjective self-confidence in knowing is influenced by the way information is presented – in the form of texts, statements, or word pairs. The highest levels of overconfidence were shown in the proposed statements; significantly lesser degrees of confidence were observed in texts learning; the lowest levels of confidence appeared in word pairs. These results may be due to the influence of logical context of the learned information (Hacker, Bol, & Bahbahani, 2008), and also due to the hard-easy effect.

The illusion of knowing depends on information length and style and is higher in larger texts. The results state that significantly higher confidence was shown while learning larger passages of information. These may be due to the influence of task performance experience on metacognitive judgements as the participants showed more efforts needed to learn larger texts. Students were also overconfident while working with the texts of the belletristic style. The reason of the higher ratings of metacognitive monitoring judgements in such texts may be because of influence of curiosity, emotional effect of information, and also the hard-easy effect.

A noteworthy finding in this study is that the illusion of knowing also depends on task type. In our study overconfidence occurred in multiple-choice questions. Thus, we can assume that subjective confidence is affected by task type.

In prospective judgements of learning the illusion of knowing had the strongest correlations with metacognitive characteristics such as metacognitive activity and metacognitive awareness. In retrospective judgements we found correlations between the indicators of the illusion of knowing and metacognitive activity, metacognitive awareness and self-confidence. Reflexivity, learning motivation, self-efficacy, and students’ introspection of fixed or changeable intellect were connected with the illusion of knowing from across the spectrum of the system of relations with metacognitive characteristics and general self-confidence.

As study of motivation is determined by a number of specific factors such as educational system, organization of the learning process, subjective characteristics of a student (e.g., age, gender, intellectual development and abilities, level of aspiration, self-esteem, and cooperation with other members of the learning process), learning motivation is significant in the increasing reliability of metacognitive monitoring (Nietfeld et al., 2005). The causes of the learning successes and failures are accounted by external and internal reasons. It is proved that those students who are governed mainly by external motivation (orientation on diploma) are characterized by overconfidence, whereas those who are guided by internal motives such as self-orientation and skills development, show underconfidence (Hacker, Bol, & Bahbahani, 2008; Kroll & Ford, 1992).

In the context of various levels of self-confidence students tend not only to overconfidence, but also to underestimation of the accuracy of tasks performance. These levels can be regarded as the indicators of ineffective metacognitive monitoring. The results that middle and low reflexive students show overconfidence may be because they do not take into account their experience of involvement in task performance situations contrary to highly and mid-reflexive students. The results are supported by the established correlations between reflexivity and metacognitive awareness.

Statistically insignificant differences of the average values of the illusion of knowing according to the notions of intellect can mean that the level of the illusion of knowing is independent of the notions of changeable intellect. In other words, the implicit theories of intellect do not significantly affect subjective confidence in the accuracy of metacognitive judgments. However, analysis of the inner-group differences in the average values ​​makes it possible to argue that in terms of changeable intellect there occurs noticeable trend towards higher performance levels of the illusion of knowing in aJOLs and aRCJs.

According to the results, students with lower levels of self-efficacy tend to demonstrate such error of metacognitive monitoring as the illusion of not knowing.

The results show that students with lower academic achievements tend towards the illusion of knowing. These results correlate with the scientific data that show the more successful people are, the less confident they are in their knowledge, and vice versa. In particular, many scientists (Dunning et al., 2003; Hacker, Bol, & Bahbahani, 2008; Jee et al., 2006; Kruger & Dunning, 1999; Miller & Geraci, 2011; Pallier et al., 2002, Wiley et al., 2005) prove that people with higher levels of knowledge tend towards lesser overconfidence (see Pasichnyk, Kalamazh, & Avgustiuk, 2017). According to Winne and Hadwin (1998), students with lower levels of academic achievements learn the information of any kind quickly, do not stop on problematic aspects, do not notice when something is unclear, and do not reread difficult for understanding passages. Savin and Fomin (2013) pay attention to direct connections between metacognitive judgements reliability and higher academic achievements in performance of different tasks. Thus, those students who demonstrate higher skills of metacognitive monitoring, receive higher marks, and, consequently, higher levels of academic achievements; students with low academic achievements tend towards overestimation of their knowledge.

However, we did not record the effects of gender and age differences on the illusion of knowing. Nevertheless, it was found that women tend towards overconfidence in their judgements. In the scientific literature there are no empirical data which state dependence of metacognitive monitoring accuracy from gender differences. In several researches attention is mainly paid to the correlations between intellect, academic achievements, motivation, and gender differences. According to McCarty and Siber (Pulford, 1996), women less tend towards overconfidence than men. Our results partly suggest the opposite (also see Pasichnyk, Kalamazh, & Avgustiuk, 2017).

The illusion of knowing is more typical for younger students, especially for those who have lower levels of academic achievements. Students with lower levels of knowledge have more difficulties with the accuracy of metacognitive judgements (overoptimistic confidence takes place here), and cannot distinguish between questions answered correctly and incorrectly. Perhaps this may be due to the fact that 17-19-year-old students, although characterized by certain maturity in mental, moral, and social terms and by conscious motives in behaviour (Shevchenko & Shevchenko, 2009), are under influence of inherent prevalence of maximalist inclinations and categorical assessments in all kinds of the learning activities. Complex and new challenges that students face from the first year of study require accurate organization of the learning process, skills of independent work with educational and scientific literature, and independent allocation of time. All these factors, apart from the development of thinking, memory capacity, and attention, also provoke generation of such processes as delaying, breaking, greater uncertainty, which, in our opinion, can cause declination of confidence in tasks performance by 20-22-year-old students.

This study had limitations that need to be mentioned. Firstly, the cross-sectional data adopted here were made in the form of the laboratory experiment, so the analyses performed represent only the results gained in this context. Thus, further research might consider the role of the illusion of knowing in metacognitive reliability in the context of real learning process. Secondly, individual psychological differences in the study of the illusion of knowing were examined only pertaining to students. That is why, further research is needed to study other social groups. Moreover, one more scope for future studies has to be taken with an aim to broaden age limitations. Qualitative methods could also enrich this type of research, permitting us to better understanding of the impact of the factors of the illusion of knowing in metacognitive monitoring reliability found in this study.

Overall, the results can imply the importance of metacognitive monitoring judgements as significant sources of how students regulate their own knowledge in the process of the learning activity. Despite some methodological limitations, the current study allows to better clarify the phenomenon of the illusion of knowing, its influence on metacognitive monitoring reliability.

A promising area of research is to provide more detailed study of the influence of the illusion of knowing not only on metacognitive monitoring, but also on metacognitive control, more thorough study of metacognitive monitoring reliability factors, and study of the illusory knowledge.

### Conclusions and Final Remarks

This research studies the illusion of knowing in metacognitive monitoring of the learning activity of university students. The analysis focuses on the effects of the different types of information proposed to learn and of personal, cognitive, metacognitive, and individual psychological characteristics of university students. These current results expand an investigation of metacognitive monitoring reliability factors. These results also have important implications for metacognitive monitoring optimization of the learning activity of university students.

## References

[r1] Avhustiuk, M. M. (2016). *Iliuzia znannia v metakohnityvnomu monitorynhu navchalnoi dialnosti studentiv vnz* [The illusion of knowing in metacognitive monitoring of the educational activity of university students] (Unpublished doctoral thesis). National University of Ostroh Academy, Ostroh, Ukraine.

[r2] Begg I. M. Robertson R. K. Gruppuso V. Anas A. Needham D. R. (1996). The illusory-knowledge effect. Journal of Memory and Language, 35, 410–433. doi: 10.1006/jmla.1996.0023

[r3] Benjamin A. S. Bjork R. A. Schwartz B. L. (1998). The mismeasure of memory: When retrieval fluency is misleading as a metamnemonic index. Journal of Experimental Psychology: General, 127, 55–68. 10.1037/0096-3445.127.1.55 9503651

[r4] Bjork, R. A. (1999). Assessing our own competence: Heuristics and illusions. In M. K. Bleckley (Ed.), *Cognitive regulation of performance: Interaction of theory and application* (pp. 435-459). 10.1002/acp.791

[r5] Bradshaw B. K. (2000). Do students effectively monitor their comprehension? Reading Horizons, 41(3), 143–154.

[r6] Brown, A. L. (1987). Metacognition, executive control, self-regulation, and other more mysterious mechanisms. In F. E. Weinert & R. Kluwe (Eds.), *Metacognition, motivation, and understanding* (pp. 65-116). Hillsdale, NJ, USA: Lawrence Erlbaum Associates.

[r7] Busey T. A. Tunnicliff J. Loftus G. R. Loftus E. F. (2000). Accounts of the confidence-accuracy relation in recognition memory. Psychonomic Bulletin & Review, 7(1), 26–48. 10.3758/BF03210724 10780019

[r8] Castel A. D. McCabe D. P. Roediger H. L. III (2007). Illusions of competence and overestimation of associative memory for identical items: Evidence from judgments of learning. Psychonomic Bulletin & Review, 14(1), 107–111. 10.3758/BF03194036 17546739

[r9] Commander N. E. Stanwyck D. J. (1997). Illusion of knowing in adult readers: Effects of reading skill and passage length. Contemporary Educational Psychology, 22, 39–52. doi: 10.1006/ceps.1997.0925

[r10] de Carvalho Filho M. K. (2009). Confidence judgments in real classroom settings: Monitoring performance in different types of tests. International Journal of Psychology, 44, 93–108. 10.1080/00207590701436744 22029451

[r11] Dotsevych T. I. (2013). Shliakhy ta zasoby diagnostyky metakohnityvnoi kompetentnosti vykladachiv [The ways and possibilities of high school teachers metacognitive competence diagnosis] Psychologis: Scientific Edition, 46(2), 62–75.

[r12] Dubovitskaia T. D. (2005). K probleme diagnostiki uchebnoi motivatsii [To the questions of the diagnosis of educational motivation] Voprosy Psykhologii, 3, 73–78.

[r13] Dunlosky J. Nelson T. O. (1992). Importance of the kind of cue for judgments of learning (JOL) and the delayed-JOL effect. Memory & Cognition, 20(4), 374–380. 10.3758/BF03210921 1495399

[r14] Dunlosky J. Rawson K. A. (2012). Overconfidence produces underachievement: Inaccurate self-evaluations undermine students’ learning and retention. Learning and Instruction, 22, 271–280. 10.1016/j.learninstruc.2011.08.003

[r15] Dunlosky J. Rawson K. A. Middleton E. L. (2005). What constrains the accuracy of metacomprehension judgments? Testing the transfer-appropriate-monitoring and accessibility hypotheses. Journal of Memory and Language, 52, 551–565. 10.1016/j.jml.2005.01.011

[r16] Dunning D. Johnson K. Ehrlinger J. Kruger J. (2003). Why people fail to recognize their own incompetence. Current Directions in Psychological Science, 12, 83–87. doi: 10.1111/1467-8721.01235

[r17] Dutke S. Barenberg J. Leopold C. (2010). Learning from text: Knowing the test format enhanced metacognitive monitoring. Metacognition and Learning, 5, 195–206. 10.1007/s11409-010-9057-1

[r18] Eakin D. K. (2005). Illusions of knowing: Metamemory and memory under conditions of retroactive interference. Journal of Memory and Language, 52, 526–534. doi:. 10.1016/j.jml.2005.01.009

[r19] Epstein W. Glenberg A. M. Bradley M. M. (1984). Coactivation and comprehension: Contribution of text variables to the illusion of knowing. Memory & Cognition, 12(4), 355–360. 10.3758/BF03198295 6503698

[r20] Fajfar, P., & Gurman, N. (2009). *When underconfidence behavior is norm: Some experimental evidences from the calibration analysis.* Paper presented at the International Association for Research in Economic Psychology (IAREP) and Society for Advancement of Behavioral Economics (SABE) Joint Conference, Halifax, Canada.

[r21] Fazio L. K. Marsh E. J. (2008). Slowing presentation speed increases illusions of knowledge. Psychonomic Bulletin & Review, 15(1), 180–185. doi:. 10.3758/PBR.15.1.180 18605500

[r22] Flannelly L. T. Flannelly K. J. (2000). Reducing people’s judgment bias about their level of knowledge. The Psychological Record, 50, 587–600. 10.1007/BF03395373

[r23] Gigerenzer G. Hoffrage U. Kleinbölting H. (1991). Probabilistic mental models: A Brunswikian theory of confidence. Psychological Review, 98(4), 506–528. 10.1037/0033-295X.98.4.506 1961771

[r24] Glenberg A. M. Epstein W. (1985). Calibration of comprehension. Journal of Experimental Psychology: Learning, Memory, and Cognition, 11, 702–718. 10.1037/0278-7393.11.1-4.702

[r25] Glenberg A. M. Epstein W. (1987). Inexpert calibration of comprehension. Memory & Cognition, 15(1), 84–93. 10.3758/BF03197714 3821493

[r26] Glenberg A. M. Wilkinson A. C. Epstein W. (1982). The illusion of knowing: Failure in the self-assessment of comprehension. Memory & Cognition, 10(6), 597–602. 10.3758/BF03202442

[r27] Griffin D. Tversky A. (1992). The weighing of evidence and the determinants of confidence. Cognitive Psychology, 24, 411–435. doi:. 10.1016/0010-0285(92)90013-R

[r28] Hacker D. J. Bol L. Bahbahani K. (2008). Explaining calibration accuracy in classroom contexts: The effects of incentives, reflection, and explanatory style. Metacognition and Learning, 3, 101–121. doi:. 10.1007/s11409-008-9021-5

[r29] Hacker, D. J., Bol, L., & Keener, M. C. (2008). Metacognition in education: A focus on calibration. In J. Dunlosky & R. A. Bjork (Eds.), *Handbook of metamemory and memory* (pp. 429-455). New York, NY, USA: Psychology Press.

[r30] Ilyina, T. I. (2003). Motyvatsia navchannia u vuzi [Motivation of education in higher educational establishment]. In V. Y. Klochko (Ed.), *Vikova Psykhologia.* Retrieved from http://medbib.in.ua/motivatsiya-obucheniya-vuze-39992.html

[r31] Jacoby, L. L., Bjork, R. A., & Kelley, C. M. (1994). Illusions of comprehension, competence, and remembering. In D. Durckman & R. A. Bjork (Eds.), *Learning, remembering, believing: Enhanced human performance* (pp. 57-80). Washington, DC, USA: National Academy Press.

[r32] Jee, B., Wiley, J., & Griffin, T. (2006). Expertise and the illusion of comprehension. In J. D. Moore & J. F. Lehman (Eds.), *Proceedings of the Annual Conference of the Cognitive Science Society* (pp. 387-392). Mahwah, NJ, USA: Lawrence Erlbaum Associates.

[r33] Juslin P. Winman A. Olsson H. (2000). Naïve empiricism and dogmatism in confidence research: A critical examination of the hard-easy effect. Psychological Review, 107(2), 384–396. 10.1037/0033-295X.107.2.384 10789203

[r34] Karpov, A. V., & Skitiaeva, I. M. (2005). *Psykhologia metakohnitivnykh protsessov lichnosti* [Psychology of metacognitive processes of the identity] (pp. 1-320). Moscow, Russia: Izdatelstvo “Instytut Psykhologii RAN.

[r35] Kashapov, M. M. (2012). Tvorcheskaia deiatelnost’ professionala v kontekste kohnitivnoho i metakohnitivnoho podkhodov [The artistic activity of the professional in the sphere of cognitive and metacognitive approaches]. In M. M. Kashapov & Y. V. Poshekhonova (Eds.), *YaRHU* (pp. 1-384). Yaroslavl’, Russia.

[r36] Kelley C. M. Lindsay D. S. (1993). Remembering mistaken for knowing: Ease of retrieval as a basis for confidence in answers to general knowledge questions. Journal of Memory and Language, 32, 1–24. 10.1006/jmla.1993.1001

[r37] King J. F. Zechmeister E. B. Shaughnessy J. J. (1980). Judgments of knowing: The influence of retrieval practice. The American Journal of Psychology, 93, 329–343. 10.2307/1422236

[r38] Klayman J. Soll J. B. Gonzalez-Vallejo C. Barlas S. (1999). Overconfidence: It depends on how, what, and whom you ask. Organizational Behavior and Human Decision Processes, 79(3), 216–247. doi:. 10.1006/obhd.1999.2847 10471362

[r39] Kolers P. A. Palef S. R. (1976). Knowing not. Memory & Cognition, 4(5), 553–558. 10.3758/BF03213218 21286981

[r40] Koriat A. (1993). How do we know that we know? The accessibility model of the feeling of knowing. Psychological Review, 100(4), 609–639. 10.1037/0033-295X.100.4.609 8255951

[r41] Koriat A. (1997). Monitoring one’s own knowledge during study: A cue-utilization approach to judgments of learning. Journal of Experimental Psychology: General, 126, 349–370. 10.1037/0096-3445.126.4.349

[r42] Koriat A. (2012). The self-consistency model of subjective confidence. Psychological Review, 119(1), 80–113. doi:. 10.1037/a0025648 22022833

[r43] Koriat A. Ackerman R. Adiv S. Lockl K. Schneider W. (2014). The effects of goal-driven and data-driven regulation on metacognitive monitoring during learning: A developmental perspective. Journal of Experimental Psychology: General, 143(1), 386–403. 10.1037/a0031768 23421442

[r44] Koriat A. Bjork R. A. (2005). Illusions of competence in monitoring one’s knowledge during study. Journal of Experimental Psychology: Learning, Memory, and Cognition, 31, 187–194. doi:. 10.1037/0278-7393.31.2.187 15755238

[r45] Koriat A. Bjork R. A. (2006). Mending metacognitive illusions: A comparison of mnemonic-based and theory-based procedures. Journal of Experimental Psychology: Learning, Memory, and Cognition, 32, 1133–1145. doi:. 10.1037/0278-7393.32.5.1133 16938051

[r46] Koriat A. Levy-Sadot R. (2001). The combined contributions of the cue-familiarity and accessibility heuristics to feeling of knowing. Journal of Experimental Psychology: Learning, Memory, and Cognition, 27, 34–53. doi:. 10.1037/0278-7393.27.1.34 11204106

[r47] Koriat A. Lichtenstein S. Fischhoff B. (1980). Reasons for confidence. Journal of Experimental Psychology: Human Learning and Memory, 6, 107–118. 10.1037/0278-7393.6.2.107

[r48] Koriat, A., Nussinson, R., Bless, H., & Shaked, N. (2008). Information-based and experience-based metacognitive judgments. In R. A. Bjork & J. Dunlosky (Eds.), *A handbook of memory and metamemory* (pp. 117-134). New York, NY, USA: Psychology Press.

[r49] Kornilova T. V. Smirnov S. D. Chumakova M. V. Kornilov S. A. Novototskaia-Vlasova Y. V. (2008). Modifikatsia oprosnikov K. Dvek v kontekste uzuchenia akademicheskikh dostizhenyi studentov [K. Dvek’s questionnaires modification in the context of the study of academic achievements of students] Psykhologicheskyi Zhurnal, 29(3), 86–100.

[r50] Kroll M. D. Ford M. L. (1992). The illusion of knowing, error detection, and motivational orientations. Contemporary Educational Psychology, 17, 371–378. doi:. 10.1016/0361-476X(92)90075-A

[r51] Kruger J. Dunning D. (1999). Unskilled and unaware of it: How difficulties in recognizing one’s own incompetence lead to inflated self-assessments. Journal of Personality and Social Psychology, 77, 1121–1134. 10.1037/0022-3514.77.6.1121 10626367

[r52] Kvidera S. Koutstaal W. (2008). Confidence and decision type under matched stimulus conditions: Overconfidence in perceptual but not conceptual decisions. Journal of Behavioral Decision Making, 21, 253–281. 10.1002/bdm.587

[r53] Maki R. H. Berry S. L. (1984). Metacomprehension of text material. Journal of Experimental Psychology: Learning, Memory, and Cognition, 10, 663–679. 10.1037/0278-7393.10.4.663 6239006

[r54] McCormick, C. B. (2003). Metacognition and learning. In I. B. Weiner (Ed.), *Handbook of psychology: Educational psychology* (pp. 79-102). 10.1002/0471264385.wei0705

[r55] McKenzie C. R. M. (1997). Underweighting alternatives and overconfidence. Organizational Behavior and Human Decision Processes, 71(2), 141–160. doi:. 10.1006/obhd.1997.2716

[r56] Merkle E. C. (2009). The disutility of the hard-easy effect in choice confidence. Psychonomic Bulletin & Review, 16(1), 204–213. doi:. 10.3758/PBR.16.1.204 19145033

[r57] Metcalfe J. (1998). Cognitive optimism: Self-deception or memory-based processing heuristics? Personality and Social Psychology Review, 2, 100–110. 10.1207/s15327957pspr0202_3 15647138

[r58] Miele D. B. Molden D. C. (2010). Naïve theories of intelligence and the role of processing in perceived comprehension. Journal of Experimental Psychology: General, 139, 535–557. doi:. 10.1037/a0019745 20677898

[r59] Miller T. M. Geraci L. (2011). Unskilled but aware: Reinterpreting overconfidence in low-performing students. Journal of Experimental Psychology: Learning, Memory, and Cognition. Advance online publication. 10.1037/a0021802 21261428

[r60] Moore D. A. Cain D. M. (2007). Overconfidence and underconfidence: When and why people underestimate (and overestimate) the competition. Organizational Behavior and Human Decision Processes, 103, 197–213. 10.1016/j.obhdp.2006.09.002

[r61] Moore D. A. Healy P. J. (2008). The trouble with overconfidence. Psychological Review, 115(2), 502–517. 10.1037/0033-295X.115.2.502 18426301

[r62] Nelson, T. O. (1999). Cognition versus metacognition. In R. J. Sternberg (Eds.), *The nature of cognition* (pp. 625-641). Cambridge, MA, USA: MIT Press.

[r63] Nelson T. O. Dunlosky J. (1991). When people’s judgments of learning (JOLs) are extremely accurate at predicting subsequent recall: The “delayed-JOL effect”. Psychological Science, 2, 267–271. 10.1111/j.1467-9280.1991.tb00147.x

[r64] Nelson T. O. Narens L. (1980). Norms of 300 general questions: Accuracy of recall, latency of recall, and feeling-of-knowing ratings. Journal of Verbal Learning and Verbal Behavior, 19, 338–368. 10.1016/S0022-5371(80)90266-2

[r65] Nelson T. O. Narens L. (1990). Metamemory: A theoretical framework and new findings. Psychology of Learning and Motivation, 26, 125–173. 10.1016/S0079-7421(08)60053-5

[r66] Nelson T. O. Narens L. Dunlosky J. (2004). A revised methodology for research on metamemory: Pre-judgment recall and monitoring (PRAM). Psychological Methods, 9(1), 53–69. doi:. 10.1037/1082-989X.9.1.53 15053719

[r67] Nietfeld J. L. Cao L. Osborne J. W. (2005). Metacognitive monitoring accuracy and student performance in the postsecondary classroom. Journal of Experimental Education, 74, 7–28.

[r68] Pallier G. Wilkinson R. Danthiir V. Kleitman S. Knezevic G. Stankov L. Roberts R. D. (2002). The role of individual differences in the accuracy of confidence judgments. The Journal of General Psychology, 129, 257–299. 10.1080/00221300209602099 12224810

[r69] Parkinson, M. M. (2009). “What did I learn?” and “How did I do?” The relation between metacognition and word learning. In P. A. Alexander (Chair), *Meta-what? Measuring Monitoring and Control.* Symposium conducted at the Annual Meeting of the American Educational Research Association, San Diego, CA, USA.

[r70] Pasichnyk I. Kalamazh R. Avgustiuk M. (2017). The illusion of knowing from perspective of metacognitive monitoring accuracy of educational activity of university students. Psychologiczne Zeszyty Naukowe: Polrocznik Instytutu Psychologii Uniwersytetu Zielonogorskiego, 1, 89–102.

[r71] Pulford, B. D. (1996). *Overconfidence in human judgment* (Unpublished doctoral thesis). University of Leicester, Leicester, United Kingdom.

[r72] Romek, V. H. (1998). Test uverennosti v sebe [Test of self-confidence]. In *Prakticheskaia Psykhodiagnostika i Psykhologicheskoie Knosultirovaniie* (pp. 87-108). Rostov-na-Donu, Russia: Ibris.

[r73] Savin E. Y. Fomin A. E. (2013). Kognitivnaia psykhologia obrazovania: auditoria kak labolatoria [Cognitive psychology of education: Class-room as laboratory]. Psykhologia v vuze*,* 3, 67-83.

[r74] Schraw G. (2009). A conceptual analysis of five measures of metacognitive monitoring. Metacognition and Learning, 4, 33–45. doi:. 10.1007/s11409-008-9031-3

[r75] Schraw G. Dennison R. S. (1994). Assessing metacognitive awareness. Contemporary Educational Psychology, 19, 460–475. doi:. 10.1006/ceps.1994.1033

[r76] Schwarzer R. Jerusalem M. Romek V. (1996). Russkaia shkala obschei samo-effektivnosti R. Schwarzera i M. Jerusalema [The Russian scale of general self-efficacy after Schwarzer and Jerusalem] Inostrannaia Psykhologia, 7, 71–76.

[r77] Serra, M. J., & Metcalfe, J. (2009). Effective implementation of metacognition. In D. J. Hacker, J. Dunlosky, & A. C. Graesser (Eds.), *Handbook of metacognition in education* (pp. 278-298). New York, NY, USA: Routledge.

[r78] Shevchenko N. F. Shevchenko A. I. (2009). Uspishnist studentiv iak problema pedahohiky vyshchoi shkoly [Students’ academic achievements as the problem of pedagogy of higher educational establishments]. Visnyk Zaporizkoho natsionalnoho universytetu*,* 2, 215-219.

[r79] Smith V. L. Clark H. H. (1993). On the course of answering questions. Journal of Memory and Language, 32, 25–38. 10.1006/jmla.1993.1002

[r80] Thiede K. W. Dunlosky J. (1994). Delaying students’ metacognitive monitoring improves their accuracy in predicting their recognition performance. Journal of Educational Psychology, 86, 290–302. 10.1037/0022-0663.86.2.290

[r81] Valdez A. (2013). Student metacognitive monitoring: Predicting test achievement from judgment accuracy. International Journal of Higher Education, 2, 141–146. doi:. 10.5430/ijhe.v2n2p141

[r82] Ward, S. B., & Clark, H. T., III. (1989). *The effect of feedback on the illusion of knowing and comprehension monitoring of college students.* Paper presented at the Annual Meeting of the Eastern Educational Research Association Savannah, GA, USA.

[r83] Whittlesea B. W. A. (1993). Illusions of familiarity. Journal of Experimental Psychology: Learning, Memory, and Cognition, 19, 1235–1253. 10.1037/0278-7393.19.6.1235

[r84] Wiley J. Griffin T. D. Thiede K. (2005). Putting the comprehension in metacomprehension. The Journal of General Psychology, 132, 408–428. doi:. 10.3200/GENP.132.4.408-428

[r85] Winne P. H. (2010). Bootstrapping learner’s self-regulated learning. Psychological Test and Assessment Modeling, 52(4), 472–490.

[r86] Winne, P. H., & Hadwin, A. F. (1998). Studying as self-regulated learning. In D. J. Hacker, J. Dunlosky, & A. C. Graesser (Eds.), *Metacognition in educational theory and practice* (pp. 277-304). Mahwah, NJ, USA: Lawrence Erlbaum Associates.

[r87] Zabrucky, K. M., Lin, L.-M., & Agler, L. (2008). Metacognition and learning. In N. J. Salkind (Ed.), *Encyclopedia of educational psychology* (2nd ed., pp. 673-676). Thousand Oaks, CA, USA: SAGE.

[r88] Zechmeister E. B. Shaughnessy J. J. (1980). When you know that you know and when you think that you know but you don’t. Bulletin of the Psychonomic Society, 15(1), 41–44. 10.3758/BF03329756

